# Current advances in the identification of plant nematode diseases: From lab assays to in-field diagnostics

**DOI:** 10.3389/fpls.2023.1106784

**Published:** 2023-01-24

**Authors:** Hudie Shao, Pan Zhang, Deliang Peng, Wenkun Huang, Ling-an Kong, Chuanren Li, Enliang Liu, Huan Peng

**Affiliations:** ^1^ State Key Laboratory for Biology of Plant Diseases and Insect Pests, Institute of Plant Protection, Chinese Academy of Agricultural Sciences, Beijing, China; ^2^ College of Agriculture, Yangtze University, Jingzhou, Hubei, China; ^3^ Grain Crops Institute, XinJiang Academy of Agricultural Sciences, Urumqi, China

**Keywords:** plant parasitic nematodes, diagnosis, PCR, Isothermal amplification, remote sensing, field detection

## Abstract

Plant parasitic nematodes (PPNs) cause an important class of diseases that occur in almost all types of crops, seriously affecting yield and quality and causing great economic losses. Accurate and rapid diagnosis of nematodes is the basis for their control. PPNs often have interspecific overlays and large intraspecific variations in morphology, therefore identification is difficult based on morphological characters alone. Instead, molecular approaches have been developed to complement morphology-based approaches and/or avoid these issues with various degrees of achievement. A large number of PPNs species have been successfully detected by biochemical and molecular techniques. Newly developed isothermal amplification technologies and remote sensing methods have been recently introduced to diagnose PPNs directly in the field. These methods have been useful because they are fast, accurate, and cost-effective, but the use of integrative diagnosis, which combines remote sensing and molecular methods, is more appropriate in the field. In this paper, we review the latest research advances and the status of diagnostic approaches and techniques for PPNs, with the goal of improving PPNs identification and detection.

## 1 Introduction

The phylum Nematoda is one of the largest in the animal kingdom, including many species and a wide variety of lifestyles. More than 25 000 nematode species are currently known (Zhang, 2013). Of these, 50% are marine salt water and 25% dwell in soil and freshwater. (Hassan et al., 2015). Over 4100 species of PPNs have been described to date (Decraemer and Hunt, 2006) representing an important constraint on global food security. They parasitize a wide range of plant species, including monocots and dicots, and are one of the most severe limiting factors for major crops, causing an estimated annual crop loss of at least 80$ billion worldwide (Nicol et al., 2011). Nematode diseases are difficult to control because their symptoms could be largely inapparent, hence, they are often overlooked. Nematode identification and differentiation can allow accurate decisions for the control of these plant parasites and the conservation of non‐parasitic nematodes.

The challenge in differentiating nematodes is not only the selection of the most accurate and suitable methods, but also due to other factors possibly effects the performance of the identification assays, such as the small size of the nematode, the high number of nematodes found in the samples, and/or the lack of particular morphological characteristics. (Floyd et al., 2002; Chitwood, 2003). The traditional classification of PPNs is based on morphological characteristics combined with morphometric values. The variations in some of these morphological and morphometric features are often conjectural, subtle, and have overlapping characteristics or show intraspecific variation that compromises accurate identification or may result in mistaken identification of species (Oliveira et al., 2011). Moreover, morphological identification is complex and time-consuming, requiring specialized and experienced researchers to be accurate (Carneiro et al., 2017).

The recent rapid development of Polymerase Chain Reaction (PCR)-based methods has facilitated their wide use for the detection and identification of PPNs. Since its invention, PCR has been one of the most prevalent and essential molecular biology methods. Currently, the PCR detection techniques applied to PPNs mainly include DNA barcoding, restriction fragment length polymorphism of the internal transcribed spacer region of ribosomal DNA (ITS-RFLP), sequence characterized amplified regions (SCAR), random amplified polymorphic DNA (RAPD), and Real-time Quantitative Polymerase Chain Reaction (RT-qPCR). Many target genes for PCR methods have been used to identify PPNs using the universal primers (**Table 1**),such as rDNA -ITS, rDNA - intergenic spacer region (IGS) (Wishart et al., 2002), 28S D2-D3 (Vallejo et al., 2021), heat shock proteins (Green et al., 2019), 18S (small subunit; SSU) (Floyd et al., 2002), and mitochondrial DNA (mtDNA) (Stanton et al., 1997). These molecular approaches compensate for the failings of traditional morphological identification to a certain extent. One or more nematode species can be detected in a mixed sample by a PCR assay, reducing the time and cost of diagnosis (Keçici et al., 2022).

**Table 1:** Some universal primer combinations used for amplification of ribosomal RNA genes of PPNs.

Primercombination and code(direction)	Primersequence(5′-3′)	Amplified region	References
G18SU(f)	GCTTGCCTCAAAGATTAAGCC	18SrRNA	(Blaxter et al., 1998)
R18Tyl1(r)	GGTCCAAGAATTTCACCTCTC		(Chizhov et al., 2006)
F18Tyl2(f)	CAGCCGCGGTAATTCCAGC	18SrRNA	(Chizhov et al., 2006)
R18Tyl2(r)	CGGTGTGTACAAAGGGCAGG		
988F(f)	CTCAAAGATTAAGCCATGC	18SrRNA	(Holterman et al., 2006)
1912R(r	TTTACGGTCAGAACTAGGG		
1096F(f)	GGTAATTCTGGAGCTAATAC	18SrRNA	(Holterman et al., 2006)
1912R(r)	TTTACGGTCAGAACTAGGG		
1813F(f)	CTGCGTGAGAGGTGAAAT	18SrRNA	(Holterman et al., 2006)
2646R(r)	GCTACCTTGTTACGACTTTT		
SSU_F_04	GCTTGTCTCAAAGATTAAGCC	18SrRNA	(Blaxter et al., 1998)
SSU_R_09	AGCTGGAATTACCGCGGCTG		
SSU_F_22	TCCAAGGAAGGCAGCAGGC	18SrRNA	(Blaxter et al., 1998)
SSU_R_13	GGGCATCACAGACCTGTTA		
SSU_F_23	ATTCCGATAACGAGCGAGA	18SrRNA	(Blaxter et al., 1998)
SSU_R_81	TGATCCWKCYGCAGGTTCAC		
designated Nem_18S_F	CGCGAATRGCTCATTACAACAGC	18SrRNA	(Floyd et al., 2005)
Nem_18S_R	GGGCGGTATCTGATCGCC		
18S-CL-F3	CTTGTCTCAAAGATTAAGCCATGCAT	18SrRNA+ ITS1-5.8S- ITS2rRNA+ 28SrRNA	(Carta and Li, 2018; Carta and Li, 2019)
28S-CL-R	CAGCTACTAGATGGTTCGATTAGTC		
18S(f)	TTGATTACGTCCCTGCCCTTT	ITS1-rRNA	(Vrain et al., 1992)
rDNA1.58S(r)	ACGAGCCGAGTGATCCACCG		(Szalanski et al., 1997)
TW81(f)	GTTTCCGTAGGTGAACCTGC	ITS1-rRNA	(Curran et al., 1994)
5.8SM5(r)	GGCGCAATGTGCATTCGA		(Zheng et al., 2000)
18S(f)	TTGATTACGTCCCTGCCCTTT	ITS1-5.8S- ITS2rRNA	(Vrain et al., 1992)
26S(r)	TTTCACTCGCCGTTACTAAGG		
F194(f)	CGTAACAAGGTAGCTGTAG	ITS1-5.8S- ITS2rRNA	(Ferris et al., 1993)
F195(r)	TCCTCCGCTAAATGATATG		
TW81(f)	GTTTCCGTAGGTGAACCTGC	ITS1-5.8S- ITS2rRNA	(Curran et al., 1994)
AB21(r)	ATATGCTTAAGTTCAGCGGGT		
D2A(f)	ACAAGTACCGTGAGGGAAAGTTG	D2-D3of28S rRNA	(Nunn, 1992)
D3B(r)	TCGGAAGGAACCAGCTACTA		
D2Tyl(f)	GAGAGAGTTAAANAGBACGTGA	D2-D3of28S	(Chizhov et al., 2012)
D3B(r)	TCGGAAGGAACCAGCTACTA	rRNA	(Nunn, 1992)
D2A(f)	ACAAGTACCGTGAGGGAAAGTTG	D2of 28S rRNA	(Nunn, 1992)
D2A(r)	GACCCGTCTTGAAACACGGA		

The advent of detection techniques for isothermal amplification, including loop-mediated isothermal amplification (LAMP) and recombinase polymerase amplification (RPA), provides additional options for the identification of PPNs. These technologies are characterized by high specificity and sensitivity. These two methods combined with the Lateral Flow Dipstick (LFD) allow for the clear visualization of the amplification products in the field, which can be identified by the naked eye (Yao et al., 2021). They also work well for field or point-of-service-based nematode detection and diagnosis. The combination of CRISPR Cas12a with RPA and LAMP methods has a detection sensitivity at the attomolar level. The specificity is enhanced by the isothermal detection technique (Gootenberg et al., 2017). CRISPR/Cas12a-based nucleic acid detection technology has been successfully used to test *Heterodera schachtii* (Yao et al., 2021), *H. avenae*, and *H. filipjevi* (Shao et al., 2022). Additionally, the development of remote sensing technology has brought new opportunities for extensive field monitoring and management of nematodes.

This article is a review of common methods for the identification of PPNs, which focuses on new isothermal amplification technologies and remote sensing methods capable of revolutionizing the approach for PPNs detection in the field.

## 2 Biochemical detection methods for PPNs

### 2.1 Isozymes analyses

Enzyme phenotyping methods, also named multifocal enzyme electrophoresis (MEE), were determined by the transport modes of isozymes, as variations in charges, molecular volumes and conformations arise from slight changes in their amino acid composition (Bogale et al., 2020). This method has the advantages of high stability, high polymorphism, and accuracy (Carneiro et al., 2017). It was first applied in the early 1970s for the identification of several common root-knot nematodes (*Meloidogyne* spp.) (Dickson et al., 1970). Many root-knot nematodes including *Meloidogyne javanica*, *M. incognita*, *M. arenaria*, *M. exigua*, and *M. paranaensis* have been identified using isozyme techniques (Carneiro et al., 2004; Carneiro et al., 2008; Muniz et al., 2008). Although this technique has been studied for other nematodes such as *H. glycines*, *Ditylenchus triformic*, and *Aphelenchus avenae* (Dickson et al., 1970), it has been best applied only for root-knot nematodes. The main reason is that certain proteins are only expressed at specific stages of the nematode life cycle, hence the isozyme extraction has strict requirements vis a vis the worm’s state (Esbenshade and Triantaphyllou, 1985; Esbenshade and Triantaphyllou, 1990). Generally, only young females can be used. Except for root-knot nematodes, young females of other plant nematode species are relatively difficult to obtain.

### 2.2 Mass spectral analyses

Matrix-assisted laser desorption/ionization time-of-flight mass spectrometry (MALDI-TOF MS) has been widely used as a diagnostic technology in laboratories for the analysis of complex molecules, by producing protein fingerprint signatures from protein extracts of organisms (Bizzini et al., 2010). MALDI-TOF MS is a highly sensitive, rapid, and reliable diagnosis method (Seng et al., 2009; Sandrin et al., 2013). Recently, researchers have discovered that MALDI biotechnology can be used for viruses, protozoa, and arthropods in addition to bacteria, mycobacteria, and fungi (Sjöholm et al., 2008; Yssouf et al., 2016; Angeletti, 2017; Vega-Rúa et al., 2018). Today, MALDI-TOF MS has also been applied for the identification of the PPNs *Anguina tritici*, *A. funesta*, *M. javanica* and *M. incognita* (Perera et al., 2005; Ahmed et al., 2011). With the increasing development of MALDI-TOF MS technology, the reduction of instrument cost, and the improvement of related databases, the technique will become a powerful tool for PPNs identification soon.

## 3 Molecular diagnosis of PPNs

### 3.1 Traditional PCR methods

#### 3.1.1 RFLP

RFLP uses restriction enzymes to either digest genomic DNA or amplified fragments, producing DNA banding patterns based on sequence divergence (Brown, 1981). The RFLP technique has the characteristics of high sensitivity, a requirement for a low amount of DNA, rapidity, and accuracy (Jarcho, 2001). The technique was first applied to the identification of nematode species by Curran et al. (1985). This method has been applied successively to identify root-knot nematodes and their physiological subspecies (Curran et al., 1986; Powers and Sandall, 1988; Zijlstra et al., 1995), *Xiphinema aameracanum* (Vrain, 1993), *Diylenchus* spp. (Wendt et al., 1993; Mahmoudi et al., 2020), *Bursaphelenchus* spp. (Aikawa et al., 2013), and *Heterodera* spp. (Zheng et al., 2003; Ou et al., 2008b; Baklawa et al., 2015). Although this method is valid in differentiating nematode isolates, it is less frequently used today owing to the complicated nature of its technique and the need for significant numbers of target DNA, usually requiring pre-culture of nematode populations (Currie et al., 2000).

#### 3.1.2 RAPD and SCAR

The RAPD method was invented by Williams et al. (1991) and is a novel genetic marker. The method involves PCR amplification of target DNA using a random sequence of 9–10 nucleotides as a primer. Polymorphism can occur due to a difference of one base in the DNA sequence from the complementary oligonucleotide primer. The use of RAPD markers for PPNs identification has the benefits of rapidity, ease, and sensitivity. Caswell-Chen et al. (1992) distinguished *H. curicifrae* from *H. schachtii* by RAPD and detected differences among six geographic populations of *H. schachtii*. Subsequently, this method was studied on both root-knot nematodes and cyst nematodes (Cenis, 1993). Because the RAPD assay is performed at a low temperature, creating a lower degree of severity for primer reductions, and replicability, in particular between laboratories. It also imposes a restriction, making it impossible to use in the field.

To compensate for the shortcomings of RAPD, it can be converted into a SCAR marker technique as proposed and applied by Paran and Michelmore in 1993. This technique not only has the characteristics of high specificity and sensitivity of the RAPD method, but has the advantages of good stability and reproducibility (Li et al., 2022). This method solves the problem of long primers and a high annealing temperature for RAPD. Fullaondo et al. (1999) transformed RAPD markers into SCAR markers to differentiate between *Globodera rostochiensis* and *G. pallida*. Subsequently, SCAR markers have been successfully used to identify *Meloidogyne* spp. (Lecouls et al., 1999; Zijlstra et al., 2000; Randig et al., 2002), *Heterodera* spp. (Ou et al., 2008a; Qi et al., 2012; Liu et al., 2014; Jiang et al., 2021), and *Bursaphelenchus* spp. (Chen et al., 2011; Feng et al., 2011).

#### 3.1.3 DNA barcoding

The DNA barcoding technique was first proposed by Hebert et al. (2003), and it uses a universal barcode to build a barcode database and analyze DNA data based on sample information to achieve identification. The advantages of this method are high primer versatility, a stable amplification system, a convenient fragment size, and low DNA sample quality requirements (Ahmed et al., 2015). DNA barcoding techniques have recently been used to study the species and phylogenetic relationships of nematodes including *Meloidogyne* spp. (Rashidifard et al., 2019), *Heterodera* spp. (Subbotin et al., 2019), and *Bursaphelenchus* spp. (Wang et al., 2015). Metabarcoding is a combination of barcoding and high-throughput sequencing (NGS). Metabarcoding was described by Taberlet et al. (2012) as the automatic identification of multiple species from a single bulk sample including several different taxa. Waite et al. (2003) used this method for community analysis of nematodes using 18S rDNA. Palomares-Rius et al. (2017) applied barcoding methods using mtDNA and rDNA regions to the phylogenetic analysis of PPNs from *Longidoridae* (Nematoda, Enoplea). There are several difficulties in the analysis of DNA metabarcoding of environmental DNA (eDNA). The eDNA is susceptible to contamination during sampling, extraction, and storage; the availability of species-specific DNA barcodes relies on the mass of the available databases. The identification of PPNs species is difficult due to the lack of available data for DNA barcoding of most known plant nematodes (Sikder et al., 2020). DNA barcoding is a tool with much potential for taxonomy. Currently, the metabarcoding technique is little utilized for PPNs detection and can be more developed in the future for PPNs identification.

#### 3.1.4 Quantitative real-time PCR (qPCR)

The fluorescent qPCR technique adds fluorescent moieties to a PCR reaction system and monitors the entire PCR process in real-time by the accumulation of the fluorescent signal. The qPCR method allows continuous monitoring of the sample during PCR using fluorescence probes or double-stranded dyes such as SYBR Green I. The method is used to quantify the unknown template by means of a standard curve. The qPCR method has the advantages of sensitivity, reliability, safety, and allowing high throughput (Smith and Osborn, 2009). A quantitative PCR technique has been developed for targeting PPNs, containing *M. enterolobii* (Kiewnick et al., 2015), *M. javanica*, *Xiphinema elongatum*, and *Pratylenchus zeae* (Berry et al., 2008), *P. penetrans* (Sato et al., 2007), *H. avenae* and *H. latipons* (Toumi et al., 2013), *H. schachtii* (Madani et al., 2005), and *H. glycines* (Goto et al., 2009; Baidoo et al., 2017). Specific technologies for PPNs identification and quantification directly from the soil or plant tissues before DNA extraction and amplification have been recently explored (Goto et al., 2009; Lopez-Nicora et al., 2012; Li et al., 2014; Jian et al., 2022). These molecular detection methods can reduce the time and labor required for identification since they eliminate the need to extract nematodes from the soil and microscopy. Although qPCR is a sensitive method for detecting low concentrations of target DNA, its use for the identification of PPNs is hampered by its cost and dependence on expensive equipment.

#### 3.1.5 Droplet digital PCR (ddPCR) technology

The concept of digital PCR was first described in 1992 by Sykes et al. (1992). It quantifies DNA molecules using a combination of the Poisson distribution and the dilution of templates to the single molecule level (Espy et al., 2006). The principle of ddPCR is to reduce a traditional PCR reaction mixture, which is like the Taqman assay, into a smaller reaction system either by diluting it in microwell plates, oil emulsion, or capillaries (Rougemont et al., 2004). It has the advantage of being very accurate at very low concentrations, with less contamination, and may be easier to sample for some diseases that are difficult to diagnose accurately (Li et al., 2018). Compared to qPCR, the ddPCR system could be used for the absolute quantitation of DNA copy numbers. The ddPCR method has high sensitivity and does not depend on a pre-enrichment for templates in extremely low concentrations. The ddPCR method has been successfully introduced into the clinic for the diagnosis of infectious diseases. Also the ddPCR has been utilized for the identification of a variety of plant pathogens including fungi, bacteria, and viruses. (Rani et al., 2019). Currently, this method has been applied to *M. enterolobii* (Chen et al., 2022).

### 3.2 Isothermal amplification technologies

#### 3.2.1 LAMP

LAMP is designed on based on automated cycling and high DNA strand replacement activity mediated by Bst polymerase. It uses 4-6 oligonucleotide primers to produce a significant amount of amplicons within 10-20min (Notomi et al., 2000). LAMP is becoming a popular assay for the detection of PPNs, because it is rapid, sensitive and easy to use in a point-of-service environment (Ahuja, 2020). The inclusion of a fluorescent dye in a positive LAMP reaction generated a color difference that enabled observation by the naked eye. (He et al., 2013). Moreover, it has also been advanced by using Lateral flow devices (LFDs) to confirm visually the existence of amplicons (Kiatpathomchai et al., 2008; Ding et al., 2010). The LAMP-LFD method allows both nucleic acid amplification and amplicon visualization to be conducted without any complex or costly equipment, which promises to enhance usability for field investigations and common field monitoring. However, the disadvantage of LAMP is that once the tube is opened, aerosol contamination can easily form, causing more serious problem of false positives. In combination with a real-time turbidimeter, LAMP results can be measured accurately and contamination can be avoided (Mori et al., 2004). With these benefits, LAMP technology has been packaged in commercially available assay kits for the testing of a diversity of pathogens which include viruses, fungi and bacteria. (Mori et al., 2001). The use of this method on PPNs has been very popular in recent years. In particular, LAMP technology has been developed for diagnosing many species of PPNs including *Bursaphelenchus* spp., *Meloidogyne* spp., *Anguina* spp., *Radopholus* spp., *Ditylenchus* spp., and *Tylenchulus* spp. (**Table 2**).

**Table 2:** The application of the LAMP technique to plant parasitic nematodes.

Genus name	Nematode species	Target region	Host	References
*Bursaphelenchus*	*B. xylophilus*	ITS-rDNA	Pine	(Kikuchi et al., 2009)
	*B. xylophilus*	ITS-rDNA	Pinus armandii var.	(Kanetani et al., 2011)
	*B. xylophilus*	Pectate lyase-3	Pine	(Kang et al., 2015)
	*B. cocophilus*	D2-D3 of rDNA	coconut and oil palm trees	(Ide et al., 2017)
*Meloidogyne*	*Meloidogyne incognita*, *M. arenaria*, *M. javanica*, *M. hapla*	ITS of rDNA	tomato	(Niu et al., 2011)
	M. enterolobii	5S rDNA-IGS2	tomato	(Niu et al., 2012)
	*M. mali*	ITS-5.8S rDNA	tomato	(Zhou et al., 2017)
	*M. chitwoodi* and *M. fallax*	IGS2-18S	tomato	(Zhang and Gleason, 2019)
	*M. partityla*	ITS-5.8S rDNA	mature pecan trees	(Waliullah et al., 2020)
*Anguina*	*A. wevelli*	ITS rDNA	—	(Yu et al., 2018)
	*A. agrostis*	ITS rDNA	—	(Yu et al., 2020)
*Radopholus*	*R. similis*	D2-D3 of rDNA	*Anthurium*	(Peng et al., 2012)
*Ditylenchus*	*D. destructor*	28S rRNA	patato	(Deng et al., 2019)
*Tylenchulus*	*T. semipenetrans*	ITS-rDNA	cirus orchards	(Lin et al., 2016)
	*T. semipenetrans*	ITS1	*citrus* rhizosphere soil	(Song et al., 2017)

#### 3.2.2 RPA

RPA is a novel, highly sensitive, isothermal DNA amplification and detection assay (Piepenburg et al., 2006). The technique is performed at 37-42°C and only requires a minimum number of DNA samples to amplify 1-10 target copies of DNA within 20 minutes (Sabate del Rio et al., 2017). RPA products can be detected by using fluorescent probes in real-time or by agarose gel electrophoresis or a lateral flow assay (Lobato and O'sullivan, 2018). The main advantages of RPA technology over other PCR detection technologies are that it is quick, sensitive, simple, and easy to use in the field. Compared to the LAMP, which needs 6-8 primers for amplification, RPA technology is simpler and requires only one pair of primers to finish amplification. RPA has been successfully applied to different species of target organisms including viruses, fungi, bacteria, animals and plants. (Lobato and O'sullivan, 2018). It has recently been reported to be highly effective in testing for a large range of PPNs including *M. javanica* (Chi et al., 2020), *M. enterolobii* (Subbotin, 2019), *M. hapla* (Subbotin and Burbridge, 2021), *B. xylophilus, M. incognita*, *M. javanica*, and *M. arenaria* (Ju et al., 2019) (**Table 3**). Although RPA has been described as highly specific, it has been reported that RPA depends on the number and distribution of mismatches in sequences of closely related DNA molecules. If one or more bases are mismatched, nematode populations cannot be differentiated based on their distribution.

**Table 3:** Information about reported studies of RPA in nematodes.

Nematode species	Target	Time (min)	Temp (°C)	Sensitivity	References
*M. enterolobii*	IGS rRNA	20	37	1/10 of a second-stage juvenile(J2)	(Subbotin, 2019)
*M. javanica*	SCAR marker	40	39	1 pg purified genomic DNA, or 0.01 adult female, or 0.1 J2	(Chi et al., 2020)
*M. hapla*	IGS rRNA	20	39	1/100 of a J2 and 1/1000 of a female	(Subbotin and Burbridge, 2021)
*M. enterolobii*, *M. incognita*, *M. javanica* and *M. arenaria*	SCAR marker	20	38	10^−2^, 10^−2^, 10^−1^, and 10^−1^ dilutions of DNA from a single J2	(Ju et al., 2019)
*H. schachtii*	RAPD marker	15-60	37	10^−4^ single cysts and single females, 4^−3^ single second-stage juveniles, and a 0.001 ng genomic DNA	(Yao et al., 2021)
*H. avenae and H. filipjevi*	SCAR marker	15	35	10^-4^ single second-stage juvenile (J2), 10^-5^ single cyst, and 0.001 ng of genomic DNA	(Shao et al., 2022)
*B. xylophilus*	ITS2	25	37	308 ± 51 of *B. xylophilus* per 10 g of pinewood	

Coupling RPA with CRISPR (Cas) systems identifies stable differences in individual bases. Cas12a and CRISPR form ribonucleoprotein, which recognizes the protospacer adjacent motif (PAM) site on the target nucleic acid and then guides the effector Cas protein to shear the target sequence. The Cas12a enzyme can non-specifically be a shear single-stranded DNA reporter-labeled fluorophore and quencher (Chen et al., 2018). It has been concluded that RPA- CRISPR/Cas12a is more sensitive and specific than RPA alone. The PPNs *H. schachtii* (Yao et al., 2021), *H. avenae*, and *H. filipjevi* (Shao et al., 2022) have been detected using RPA-CRISPR/Cas12a technology. The combination of LAMP and CRISPR/Cas12a can also be used for pathogen detection. The use of Cas12a is a powerful method for virus detection (Broughton et al., 2020) set up a DETECTOR platform that combined RT-LAMP and CRISPR/Cas12a for SARS-CoV-2 diagnosis. In the future, this technique could also be applied to the detection of PPNs.

## 4 Direct detection of PPNs in the field

In order to truly implement field testing, the feasibility of field operation encompassing the entire detection process must be considered, including sample handling, the amplification process, and visualization of the results. In a previous study, isolating nematodes from a Baermann funnel or directly picking nematodes from plant root galls was time-consuming and required specialized techniques. DNA could be extracted directly from plant root nodules using the Flinders Technology Associates (FTA) technique, reducing the cost and time for diagnosis by simplifying sample storage, transport, and extraction. All of the steps of FTA-based archiving and DNA preparation are carried out at room temperature, which significantly reduces the expense and is environmentally friendly (Marek et al., 2014). FTA technology has been used for the DNA extraction of *D. dipsaci*, *H. schachtii*, and *M. hapla* (Marek et al., 2014; Peng et al., 2017). The drawback of this method is that it is limited to the extraction of pathogenic DNA from plant tissues and cannot be utilized for the extraction of DNA from soil or other media. Commercial kits for direct extraction of nematode soil DNA have now been developed and used successfully in several laboratories. The use of these kits also saves the time consumed by nematode isolation and the cost of instruments. Using only a small amount of DNA in this template, the target nematode can be detected. Soil DNA, including that from *Pratylenchus neglectus*, *P. thornei*, *M. incognita, R. similis*, and *H. schachtii*, was extracted using soil kits for the successful detection of these nematodes (Yan et al., 2008; Hu et al., 2011; Min et al., 2011; Peng et al., 2012; Jiang et al., 2021; Yao et al., 2021). Although this method has several advantages such as time-saving, simplicity, and efficiency, the soil kit can extract no more than 10 g of soil at a time. The uneven distribution of nematodes makes it difficult to extract DNA containing the target nematodes. This problem might be solved by repeating the assay multiple times to improve the detection rate for nematodes. In the amplification stage, using RPA and LAMP techniques or these two methods combined with CRISPR/Cas12a allows DNA amplification in 15-60 min without thermal cycling and expensive instruments (i.e., PCR instruments or fluorescence PCR instruments) compared to conventional PCR. It offers the possibility of field application for PPNs detection. The results of a combination of LFD technology and these methods are visible to the naked eye. RPA combined with the CRISPR/Cas12 assay has been applied to the detection of the PPN *H. schachtii* in the field (Yao et al., 2021), *H. avenae*, *and H. filipjevi* (Shao et al., 2022). Therefore, the combination of the FTA technique, the kit method for soil sample extraction, and a combination of LFD technology and RPA/LAMP-CRISPR/Cas12 can fully and truly realize the field detection of nematodes.

## 5 The remote sensing method for PPNs

Remote sensing is a method of observing and acquiring information about the properties of the studied entity without physically coming in contact with it (Kundu et al., 2022). The method could determine the presence of a nematode species by the change of symptoms in the above-ground parts of a plant. It avoids damage to the host and saves time and cost of diagnosis. Remote sensing is a fast, non-invasive, and highly effective process of acquiring information that has a wide coverage. Various spectroscopic and imaging approaches have been performed for the detection of PPNs, such as visible, multiband, infrared, and fluorescence spectroscopy, fluorescence imaging, multispectral and hyperspectral imaging, thermography, and nuclear magnetic resonance spectroscopy. Norman and Fritz (1965) were the first to use infrared sensors for pre-sign detection of *R. similis* in citrus trees. Subsequently, *R. reniformis* was detected by Heald et al. (1972) using airborne infrared imaging methods in cotton fields. Heath et al. (2000) predicted the amount of the nematodes *G. rostochiensis* and *G. pallida* on potatoes based on non-destructive hyperspectral measurements with a combination of GIS and RS technologies. Remote sensing coupled with GIS technologies was employed to identify and quantify an *H. glycines* population (Nutter et al., 2002). Three data preprocessing approaches were tested to evaluate their suitability for detecting *H. schachtii* and *R. solanii* (Hillnhutter et al., 2012). Pine wood nematode disease was discovered by Pan et al. (2014) based on hyperspectral remote sensing technology. Three methods, visible light imaging, thermometry and spectroscopy, were compared for their ability to detect *H. schachtii* in two sugar beet varieties (Joalland et al., 2017). Currently, remote sensing techniques have accuracy issues, as some nematodes are misdiagnosed due to similar symptoms and a lack of sufficient survey data for nematode surveillance modeling.

## 6 Machine learning for PPNs identification

Machine Learning or Artificial intelligence (AI) is a novel technology for nematode identification and quantitation based on image analysis (Bogale et al., 2020). It is an effective method for processing a large number of samples and identifying unique and minute items such as nematodes eggs and cysts in a complex background (Akintayo et al., 2018). Biological image datasets for multiple genera of PPNs were established and used to identify them based on the deep convolutional neural networks (CNNs) method (Lu et al., 2021). A convolutional CNNs model for identification of nematodes in soybean crop was developed by Abade et al. (2022).AI or Deep learning combined with hyperspectral image analysis is more popular because of the advantages this assay presents over direct soil methods (Arjoune et al., 2022). A combination of infrared spectra analysis and AI assay was used to detect rootknot nematode *M. enterorlobii* at the early stage of infection (San-Blas et al., 2020). AI, a relatively new technology, is gradually being applied to the field of PPNs detection. Though the technique could overcome the drawbacks of reduced specialist and subjective judgment, the generation of sufficient data may become a bottleneck in the development of AI.

## 7 Conclusion and future perspectives

**Figure 1:**
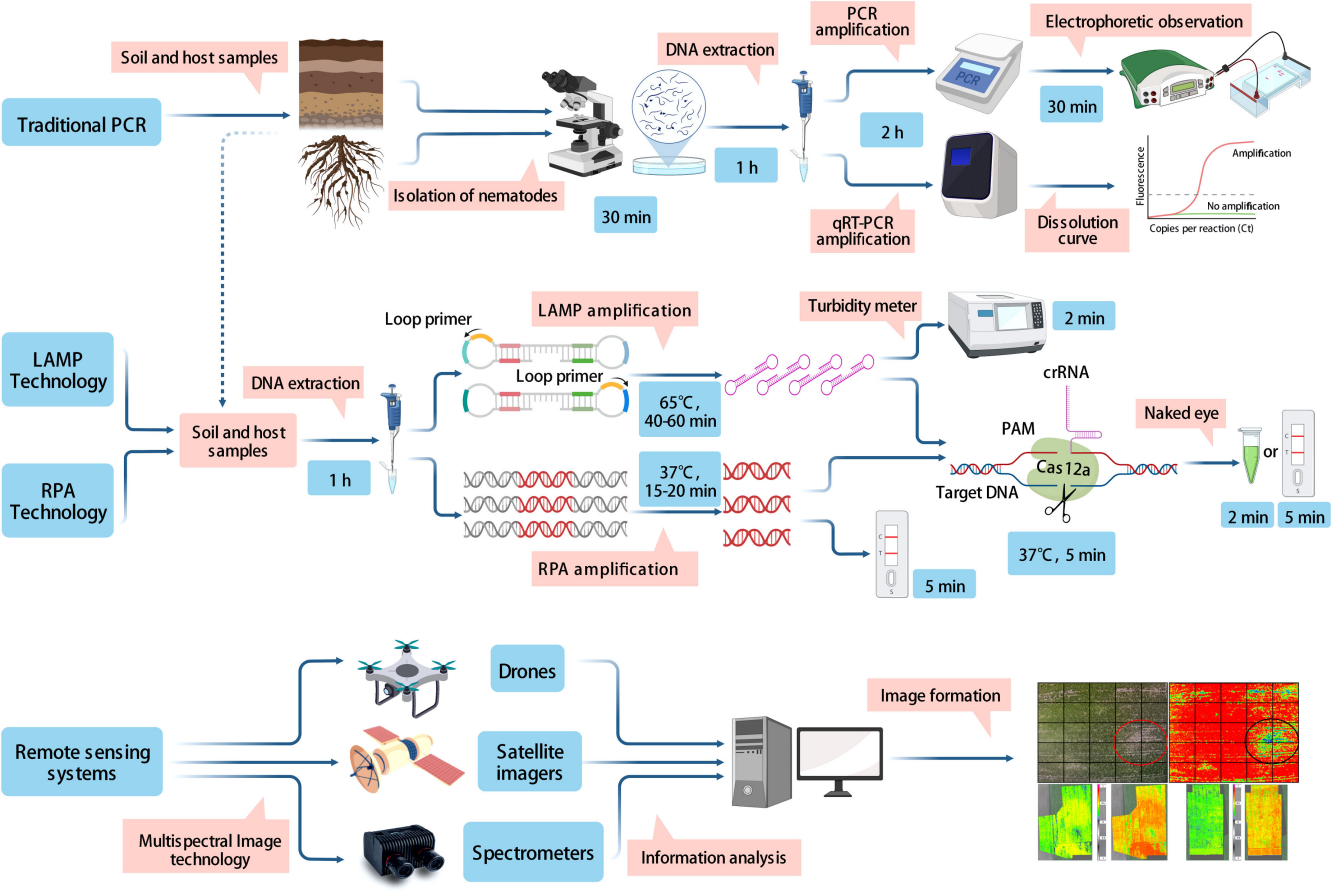
Demonstration of the principles and working processes involved in traditional PCR methods, isothermal amplification techniques, and remote sensing techniques.

In this article, we reviewed various existing methods for the detection and diagnosis of PPNs, such as morphological and biochemical methods, traditional PCR, isothermal amplification technologies, and remote sensing techniques. Practically, no single method or technique exists for diagnosing PPNs. Each approach has its strengths and weaknesses, therefore, we concluded the characteristics of each method (**Table 4**). Morphology-based classification forms the foundation of taxonomy, but morphological and morphometric characters are subtle and subjective, which may lead to inaccurate identification of a species (Floyd et al., 2002; Chitwood, 2003). In the future, the Protein-based approach play an important role in studies of species identification. However, the complexity of protein expression patterns and the ease of degradation of extracted proteins may affect the accuracy of the assessment; this restriction is the major challenge in the use of this technique. PCR molecular marker technologies have been widely used for PPNs detection, which compensate for the lack of morphological identification. The representative PCR, ddPCR, and qPCR technologies use dynamics of denaturation that drive replication events in control, and show excellent testing capability (Agüero et al., 2003; Mika et al., 2020; Wang et al., 2020). However, the requirements for expensive equipment and lack of trained scientist lead to their restriction for use in the laboratory and field detection. The isothermal amplification method is suitable for field testing because it does not require a device for temperature loop control (Niemz et al., 2011). The isothermal amplification technique takes much less time and cost than conventional PCR amplification. Among isothermal amplification techniques, LAMP-LFD and RPA-LFD are quickly evolving in the field of identification, because of their obvious specificity, efficiency, and visualization (**Figure 1**). The previously mentioned techniques are only useful for identifying small-scale samples, but remote sensing techniques could be quickly applied to detect large infected areas in the field employing various instruments such as drones, spectrometers, and satellite imagers. Remote sensing technology has contributed greatly to the prediction of damage caused by PPNs in the field.

**Table 4:** Comparison of different plant nematode detection methods.

Category	Technology	Advantages	Disadvantages	Site	References
Morphology	Morphological methods	Intuitive, low cost	Difficult to judge accurately; complex to operate and requires specialized technicians	In the lab	(Oliveira et al., 2011)
Biochemical Methods	Isozymes	It can reflect phylogenetic relationships; High sensitivity	Mainly used only for root-knot nematodes; Time-consuming		(Dickson et al., 1970)
	Mass spectral analyses	fast, reliable, high sensitivity	Time- consuming, requires specialized skills		(Rivero et al., 2022)
PCR Methods	DNA barcoding	Accuracy	Time-consuming		(Hebert et al., 2003)
	Droplet digital PCR	High sensitivity and low amount of template DNA	Expensive reagents and instruments		(Rougemont et al., 2004)
	Gene chip technology	Fast, accuracy	Expensive equipment, immature technology		(Fodor, 1997)
	RFLPs	Reliable and reproducible	Complex operations, requiring large amounts of DNA		Blok and Powers, 2009
	RAPD	Generates a large amount of information	Lacks repeatability, requires strict experimental reaction		(Feng et al., 2005)
	SCAR	High sensitivity and specificity	Time-consuming		(Chen et al., 2011)
	RT-qPCR	Sensitive, reliable	Time- consuming, equipment relatively expensive		(Berry et al., 2008)
	ddPCR	High sensitivity, Simple, convenient	Expensive instruments		Chen et al., 2022)
Isothermal Amplification Technology	LAMP	Low cost, simple operation, low equipment demand	False-positive results	Outdoors and in the field	(Ahuja and Somvanshi, 2021)
	RPA	Fast, high sensitivity and specificity, Low cost, simple operation, low equipment demand, visualization of results	False-positive results; Required to design specific primers, probes, and gRNA		(Babu et al., 2018)
	LRPA- CRISPR/Cas12a				
Spectral techniques	Remote sensing systems	Fast, large-area detection, dynamic monitoring	Requires technical personnel expertise, difficult to capture detailed changes		(Tao et al., 2020)
Machine Learning	Artificial intelligence	fast, accurate and eliminate human errors	Lack of professional classification experts and a sufficient number of databases	In the lab	(Almalki, 2022)

In the field, rapid and accurate early diagnosis of PPNs is essential to control nematode damage. PPNs mainly damage the root tissues of plants, symptoms on aboveground parts are often not apparent, and are difficult to differentiate with the naked eye unless the damage is particularly serious. At the early stage of a nematode infection, no obvious changes are evident in the aboveground parts of the plant. However, hyperspectral methods can find significant differences in the leaf area index, absorptivity, photosynthetically active radiation, or canopy depression. This may be due to changes in the chlorophyll content of the above-ground parts of the plant, causing a change in the spectrum of the host plant (Din et al., 2017). Thus, the first use of remote sensing technology would be a prediction of the presence of the location of nematode infestation in the field. While remote sensing techniques face the problem that many nematode symptoms (i.e., wavelength, lutein, and chlorophyll, etc.) are similar, it is difficult to capture these changes in detail, leading to misjudgment. To solve this problem, RPA/LAMP-LFD or RPA/LAMP-CRISPR/Cas and other detection methods can be used to accurately survey the samples in a potential occurrence area. Time and economic losses caused by the blind application and ineffective use of nematicide can be avoided. It is noteworthy that in the field environment, every step from sampling and nucleic acid extraction to obtaining test results is exposed to the risk of contamination. Therefore, the integration of sample pretreatment, target identification and signal acquisition into a single device to establish an integrated nucleic acid detection system is a major development trend for future pathogenic nematode detection.

## Author contributions

Conceptualization, HP, DP, and EL; Article framework, L-aK, WH, and CL; software, HS and PZ; Literature collection, HS, CL, and PZ; writing—original draft preparation, HS and PZ; writing—review and editing, HP and EL; project administration, DP; funding support, HP. All authors contributed to the article and approved the submitted version.
